# Molecular Characterization and Comparative Genomic Analysis of vB_PaeP_YA3, a Novel Temperate Bacteriophage of *Pseudomonas aeruginosa*

**DOI:** 10.3389/fmicb.2020.00947

**Published:** 2020-06-03

**Authors:** Xinyan Yu, Jing Xu, Yu Gu, Ruiyang Zhang, Yefei Zhu, Xiaoqiu Liu

**Affiliations:** ^1^Key Laboratory of Pathogen Biology of Jiangsu Province, Department of Microbiology, Nanjing Medical University, Nanjing, China; ^2^Laboratory Medicine Center, The Second Affiliated Hospital of Nanjing Medical University, Nanjing, China

**Keywords:** *Pseudomonas aeruginosa*, temperate phage YA3, genome analysis, structural proteome, integration site, phylogenetic analysis

## Abstract

It is well known that bacteriophages play crucial roles in many aspects, such as controlling the number and the diversity of bacteria and participating in horizontal gene transfer, which is a key process in the evolution of bacteria. However, so far, the number of temperate bacteriophages is still limited, and their life processes are severely unknown, except for members of the lambdoid family of coliphages. In this study, a novel temperate phage of *Pseudomonas aeruginosa*, YA3 (vB_PaeP_YA3), was isolated from waste water. The morphology of YA3 suggested that it is a *Podoviridae*. The YA3 genome is a circular double-stranded DNA of 45,253 bp, with an average G + C content of 57.2%. A total of 65 open reading frames (ORFs) were predicted according to the sequence of YA3’s genome, of which only 32 (49.2%) ORFs were assigned with putative functions and 13 ORFs were confirmed by the structural proteome. Genome and proteome analyses confirmed the lysogenic nature of this phage, which encodes the typical lysogen-related proteins integrase, CI, Cro, and Q protein. The genome of YA3 is most closely related with that of temperate phage vB_PaeP_Tr60_Ab31, whereas the homology coverage is just 48%. There are many critical differences between their genomes, involving promoters, lysis pathways, and regulation patterns. YA3 is capable of stably lysogenizing its host *P. aeruginosa* PA14, targeting the integration site within the serine tRNA gene (PA14_RS20820), which is similar with phage vB_PaeP_Tr60_Ab31. The phylogenetic analysis is more complicated than we thought. Based on phage terminase large subunit (TerL) and CI proteins, phage YA3 is related with phage lambda, while their genome coverage is extremely low (<1%). Therefore, phage YA3 is a considerably novel lambda-like temperate phage, and a further study of its genome may deepen our understanding of the interaction between lysogenic phages and their bacterial hosts.

## Introduction

*Pseudomonas aeruginosa* (*P. aeruginosa*) is one of the most common opportunistic human pathogens with a high diversity. Because of its broad ecological range, *P. aeruginosa* can colonize various habitats, including soil, water, and the animate surfaces of plants, animals, and humans. By forming biofilms, it can even adhere strongly to the surface of medical devices—breathing tubes and catheters, for example, which makes it an important bacteria that cause nosocomial infections (Crull et al., 2018). As a result, *P. aeruginosa* can cause many kinds of chronic and acute life-threatening infections, such as burn infection, cystic fibrosis, otitis media, and septicaemia, especially in immunocompromised patients (Hilker et al., 2015). Furthermore, *P. aeruginosa* owns multiple resistance mechanisms to antibiotics, so its drug resistance rate is extremely high and has been growing rapidly due to the plentiful use of antibiotics (Hauser, 2009; Zowalaty et al., 2015). The emergence of multidrug-resistant and pan-drug-resistant *P. aeruginosa* strains makes the treatment of its infection a thorny problem.

In the lysogeny cycle, temperate phages can participate in horizontal gene transfer (Touchon et al., 2017) and regulate bacterial host behavior throughout lysogenic conversion and interactions between them (Argov et al., 2017). It is reported that the presence of prophages can increase bacterial fitness and ecological competitiveness (Abedon, 2008; Fortier and Sekulovic, 2013). Besides that, the pathogenic toxins of bacteria such as *Corynebacterium diphtheriae*, *Staphylococcus aureus*, and *Escherichia coli* are encoded by phages (Harald et al., 2004; Ben et al., 2013). The transcription factor Cro of lambda-phages in Enterohemorrhagic *E. coli* (EHEC) can activate the Enterohemorrhagic EHEC type III secretion system (T3SS) during lysogeny (Hernandez-Doria and Sperandio, 2018). A temperate phage can neutralize the expression of a virulence factor by integrating itself into the bacterial genome in the mammalian gut (Hsu et al., 2020). The infection of *P. aeruginosa* strain PAO1 (serotype O5) by *Pseudomonas* temperate phage D3 (one of the homologous phages of YA3) causes the serotyping switch of *P. aeruginosa* serotype O5 to O16 (Kropinski, 2000). In *P. aeruginosa*, prophages were also proven to promote the virulence of *P. aeruginosa*, and their lytic induction could lead to biofilm formation and regulate bacterial densities at the site of infection (James et al., 2015). In addition, it is believed that temperate phages could promote the adaptive evolution of bacterial pathogens (Davies et al., 2016; Weitz et al., 2017). Therefore, the impact of temperate phages on their hosts could be versatile, and interactions between them are extremely complex.

At present, only 9,083 phage genome sequences are available from the National Center for Biotechnology Information (NCBI) database, 325 of which are sequences of *Pseudomonas* phages (as of 14-January-2020). Obviously, the number of isolated phages is highly limited. In addition, the vast majority of them are lytic, and the number of lysogenic phages is even less. Furthermore, as for the obtained phages, we only know very small part of the genes’ functions of phages, which are mainly related with morphology and the lysis process. The genes responsible for regulating the interaction between phages and their hosts are severely unknown. Hence, the isolation of new temperate phages is needed, and the analysis of their genomes and life process will lead to a better comprehension of relationship and co-evolution between phages and bacterial hosts.

Here we introduced a new temperate phage, YA3, and performed the evolutionary analysis. We demonstrate that YA3 is a novel temperate phage of *P. aeruginosa* and a new member of the lambdoid family. The isolation and an in-depth study of the new lambdoid phage, YA3, will contribute to further understanding the evolutionary relationship between temperate phages and their hosts.

## Materials and Methods

### Bacterial Strains, Media, and Growth Conditions

The bacterial strains used in this study are listed in Supplementary Table S1. *P. aeruginosa* PAO1, *P. aeruginosa* PA14, and *E. coli* MG1655 are the stock strains of our lab. Twenty clinical *P. aeruginosa* strains, which are all antibiotic resistant, were isolated from the clinical samples of patients in the Second Affiliated Hospital of Nanjing Medical University, Nanjing, China. Seventeen out of the 20 isolates were resistant to at least two of the anti-pseudomonal antibiotic classes tested. All the strains were cultivated in Luria–Bertani (LB) medium at 37°C.

### Isolation and Propagation of Bacteriophages

*Pseudomonas aeruginosa* strain PA14 was used for phage isolation and amplification from wastewater in Nanjing. In brief, the sewage samples were filtered using 0.22-μm-pore filters (Millipore, United States) to remove the bacteria. Then, the filtrate was added to the *P. aeruginosa* PA14 culture in early log-phase, shaken, and cultured at 37°C for 24 h to enrich the phages. The culture was then centrifuged at 12,000 *g* for 20 min at 4°C. The supernatant was tested for plaque formation with *P. aeruginosa* PA14 using the double-layer agar plate method (Ellis and Delbruck, 1939). Single plaques on the double-layer plate were picked to start a new round of amplification. The infection cycle was repeated until the plaques were homogeneous. The phages were then amplified and stored at 4°C.

### Purification of Phage YA3

The purification of phage YA3 was performed as described previously with slight modifications (Heo et al., 2009). Briefly, *P. aeruginosa* PA14 culture at the early log-phase (OD_600_ = 1.0) was infected by phage YA3 with shaking at 37°C for 24 h. The cell debris was removed by centrifugation (14,000 *g*, 30 min, 4°C) and the supernatant was passed through 0.22-μm-pore filters. The filtered supernatant was concentrated by ultracentrifugation (100,000 *g*, 2 h, 4°C) to precipitate the phage, and then the pellet was suspended in SM buffer (5.8 g/L NaCl, 2 g/L MgSO_4_⋅7H_2_O, 50 mM Tris-HCl, pH 7.5). The concentrated suspension was further purified by cesium chloride gradient centrifugation (90,000 *g*, 20 h, 4°C). The phage zone was collected and diluted using SM buffer and then ultracentrifuged (210,000 *g*, 3 h, 4°C) to remove CsCl. Finally, the pellet was resuspended using SM buffer to yield the highly purified phage.

### Transmission Electron Microscopy

The highly purified phage sample was dropped onto a carbon-coated copper grid for 3 min, negatively stained with 2% (w/v) phosphotungstic acid for 3 min, and examined using a FEI Tecnai G2 Spirit Bio TWIN transmission electron microscope at 80 kV.

### Host Range Analysis of YA3

The killing range of YA3 against the different tested strains was determined using the standard spot tests (Kutter, 2009). Then, 3 ml of melted soft agar (0.6% agar) was added to 100 μl of overnight bacterial cultures, which were then mixed and overlaid on LB plates. After solidification, 5 μl of each serially diluted phage YA3 stock suspensions (10^10^–10^2^ pfu/ml) was dropped onto the plates containing each tested strain. Following overnight incubation at 37°C, the attenuation of bacterial growth was examined to assess the bacterial sensitivity to YA3. All the experiments were conducted according to the standard institutional guidelines of Nanjing Medical University (Nanjing, China). The study was approved by the research and ethics committee of the Second Affiliated Hospital of Nanjing Medical University, and informed consents were obtained from all the patients.

### Extraction, Sequencing, and Analysis of YA3 Genome

The purified phage sample was treated with DNaseI (New England Biolabs) and RNaseA (Tiangen Biotech) at 37°C for 2 h to digest the exogenous DNA and RNA, followed by digestion with proteinase K (Tiangen Biotech) at 55°C for 15 min. The phage genome DNA was further prepared with a TIANamp Bacteria DNA Kit (Tiangen Biotech). Restriction enzyme digestion of the genome DNA was performed according to the manufacturer’s instructions (Fermentas). YA3 genome was sequenced using an Illumina HiSeq 2500 sequencer, and reads were assembled into a whole genome using SOAPdenovov2.04 and GapCloserv1.12 software.

Putative open reading frames (ORFs) were predicted using Artemis software^1^, with a threshold of 30 amino acids (aa) as the minimum length of proteins. Function annotation of the predicted proteins was carried out based on the BLAST results at NCBI^2^ against the non-redundant protein sequence database and the HHpred analysis (Johannes et al., 2005; Zimmermann et al., 2018). For protein motif searching, the Conserved Domain Search Service (CD Search) at NCBI^3^ was used. The ExPASy Compute pI/Mw tool was used to calculate the molecular masses and the isoelectric points of the putative proteins. In addition, transfer RNAs (tRNAs) were searched using tRNAscan-SE^4^ (v1.23), and ribosome RNAs (rRNAs) were identified using RNAmmer^5^ (v1.2). The phage promoters were predicted using the PhagePromoter server^6^ (Sampaio et al., 2019) and the BDGP prediction program^7^. The rho-independent terminators were searched using ARNold^8^, which combines two complementary programs, Erpin (Gautheret and Lambert, 2001) and RNAmotif (Macke et al., 2001). The whole viral nucleotide sequence similarities between phages were determined using megablast at NCBI. The Easyfig program was used to compare the arrangements and the similarities between different phages (Sullivan et al., 2011). The global alignment of putative amino acid sequences was carried out using the EMBOSS Needle tool at the European Molecular Biology Laboratory-European Bioinformatics Institute (EMBL-EBI). Phylogenetic analyses between the genomes of related phages were performed with MEGA using the neighbor-joining algorithm.

### Structural Protein Analysis of YA3

The highly purified phage particles were separated by sodium dodecyl sulfate polyacrylamide gel electrophoresis (SDS-PAGE) using 12% acrylamide concentration. The gels were stained with silver as described by Shevchenko et al. (1996). For protein identification by liquid chromatography-electrospray ionization with tandem mass spectrometry, the phage particles were digested with trypsin, and the tryptic peptides were analyzed by a Q Exactive mass spectrometer (Thermo Scientific, United States). The corresponding ORFs were searched using the MASCOT engine (Matrix Science, London, United Kingdom; version 2.2) against the protein sequence library of phage YA3.

### Isolation and Genome Sequencing of YA3-Lysogenized PA14 Strain

YA3-lysogenized *P. aeruginosa* PA14, named PA14-YA3, was isolated using the following method. Phage YA3 was plated with its host strain PA14 using the double-layer agar plate method. After more than 48 h of incubation, the cells were picked from the turbid area of plaques and streaked onto a fresh LB solid plate. The colonies were screened for lysogenization by PCR using four different pairs of primers (Supplementary Table S2) within the YA3 genome sequence. Then, the positive strain was subcultured and identified by PCR for five rounds to ensure the stability of lysogenization.

The genomic DNA of PA14-YA3 was extracted with a TIANamp Bacteria DNA Kit (Tiangen Biotech) and sequenced using Illumina HiSeq technology. The reads were assembled using ABySS v2.0.2 and GapCloser v1.12. Gene prediction was performed by Glimmer3, and functional annotation was carried out by blast against non-redundant (NR in NCBI) database. In addition, tRNAs and rRNAs were identified using tRNAscan-SE v1.23 and RNAmmer v1.2, respectively. The scaffolds of lysogenized PA14 were aligned with the phage YA3 genome sequence using two-sequence blast to identify the region related with phage YA3 and determine the phage integration site.

### Nucleotide Sequence Accession Number

The nucleotide sequence of phage YA3 genome has been deposited in GenBank under accession number MK819239.

## Results and Discussion

### Isolation and Characteristics of the YA3 Bacteriophage

**FIGURE 1:**
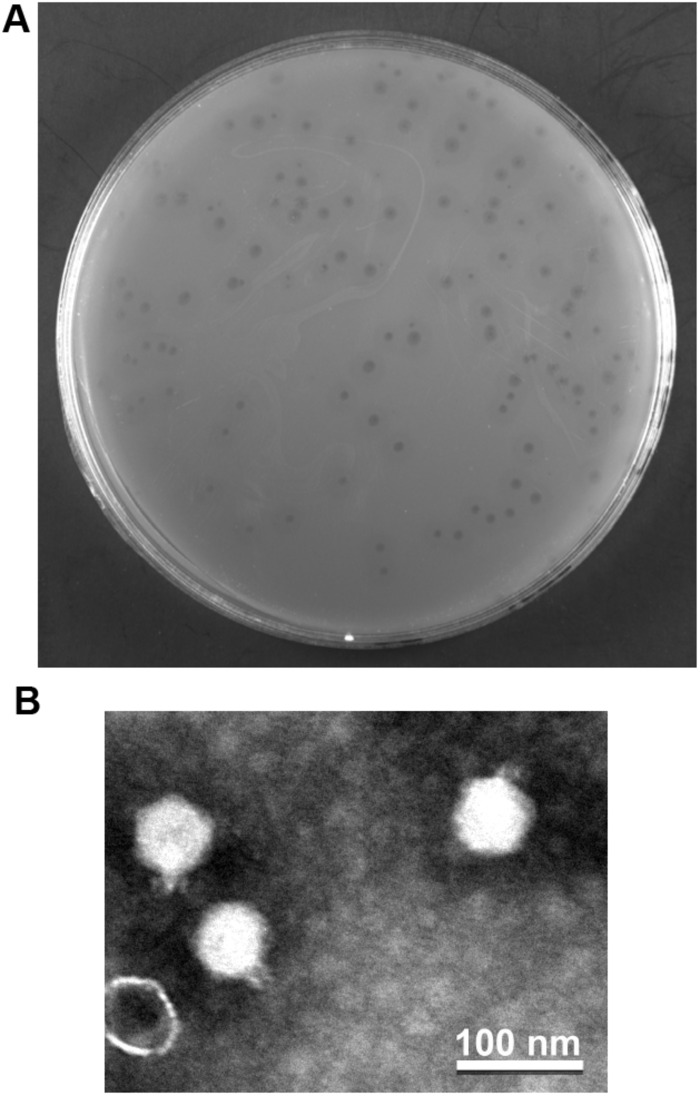
Isolation and morphology of phage YA3. **(A)** Plaque morphology of phage YA3 on *Pseudomonas aeruginosa* strain PA14. **(B)** Transmission electron micrographs of phage YA3. The scale bar represents 100 nm.

The phage vB_PaeP_YA3 (referred to herein as YA3) was isolated from the polluted river in Nanjing, using *P. aeruginosa* PA14 as the host strain. As shown in Figure 1A, YA3 produced turbid round plaques on the lawn of *P. aeruginosa* PA14 after 16 h at 37°C. Transmission electron microscopy analysis showed that YA3 had an icosahedral head, with a diameter of approximately 55–60 nm, and a short tail, with a length of approximately 10 nm (Figure 1B). This morphology suggested that YA3 is a member of the *Podoviridae* family.

The host range of phage YA3 was also determined. The results showed that YA3 only infects *P. aeruginosa* PA14 strain but not *P. aeruginosa* PAO1 or *E. coli* MG1655 (Supplementary Table S1). For the 20 clinical antibiotic-resistant isolates of *P. aeruginosa* from different sources, YA3 infects none of them. These data indicated that YA3 has a very narrow host range and may selectively infect its host. It was previously reported that the infection specificity of phages is extremely complicated and dependent on many details at every step of the phage life cycle (De Jonge et al., 2019). Among them, the attachment stage is likely the most important step. Phages that bind to a single receptor of host bacteria tend to have a narrow host range, while those binding to multiple different receptors may be capable of infecting more diverse hosts. Hence, phage YA3 may bind to a single receptor specifically, and the following infection processes may also be complex and refined.

### Sequencing and Annotation Analysis of the YA3 Genome

**FIGURE 2:**
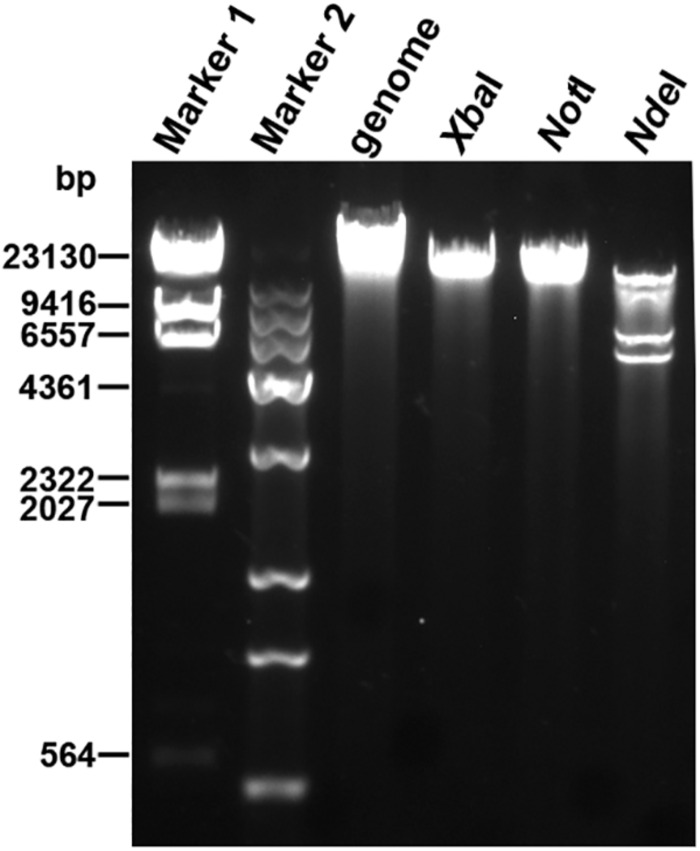
Restriction digestion profiles of the YA3 genome. The genome of YA3 was digested with *Xba*I, *Not*I, and *Nde*I. Marker 1: λDNA/*Hin*dIII marker. Marker 2: *Trans* 15K DNA ladder marker.

Next-generation sequencing was performed to determine the whole-genome sequence of phage YA3. The complete genome of YA3 is 45,253 bp, and its G + C content is about 57.2%, which is lower than that of *P. aeruginosa* PA14 (G + C 66.3%), a common feature of *P. aeruginosa* phages (Kwan et al., 2006). In order to confirm the obtained nucleotide sequence, the purified YA3 genome was further digested with *Xba*I (one cut site on YA3 genome), *Not*I (one cut site on YA3 genome), and *Nde*I (four cut sites on YA3 genome) (Figure 2). We observed a single band from the restriction digestion with *Xba*I or *Not*I and four bands from *Nde*I, respectively. Therefore, the YA3 genome is supposed to be a circular double-stranded DNA, but the DNA of the tailed phages to be packaged is generally in the form of concatemers and packaging of the DNA is accompanied by DNA cutting to produce unit-length linear chromosomes (Casjens, 2011). Terminases staggered nicks at an adjacent site to generate the cohesive ends of mature phage chromosomes (Casjens, 2011). Because the YA3 genome encoded typical terminase, it should use a similar protocol to package linear genome DNA in particle, but the results of endonuclease reaction indicated that the YA3 genome is a circular DNA. We conclude that the YA3 genome should be a linear DNA, which may be circularized through the putative cohesive end inside of phage particles or in the process of DNA purification. Our further experiment will focus on this.

**FIGURE 3:**
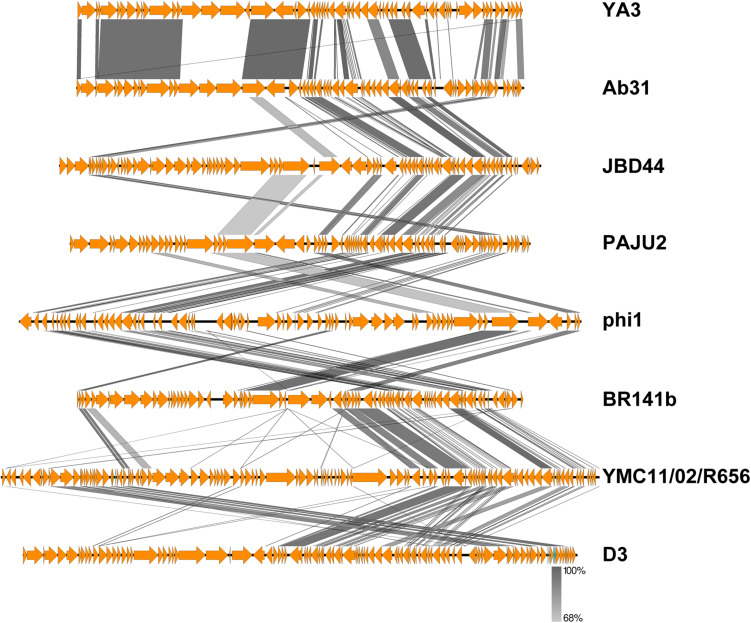
Comparative genomic analysis of phage YA3 and other seven homologous phages. Open reading frames of phage genomes are presented in orange, and the amino acid identities of homologous proteins are shown with different gray colors.

To further understand the YA3 genome, we performed a megablast using its whole nucleotide sequence at the NCBI website. The result showed that only seven complete genomes of phages, which all infect *Pseudomonas* strains, have more than 5% coverage with the YA3 genome (Table 1). Phage vB_PaeP_Tr60_Ab31 (subsequently called Ab31) exhibits the highest homology coverage, which is about 48%. The sequence coverage of all the other six phages with YA3 is below 15% (Table 1). Besides that, of these seven phages, only the phage Ab31 genome is circular, and it possesses the morphology of a podovirus, which are characteristics similar with YA3. We then used Easyfig software to compare the genomic sequences of YA3 and the seven related phages (Figure 3). In accordance with the blastn analysis result, YA3 is most closely related with Ab31. Furthermore, the similar regions between them are segmented, indicating the occurrence of horizontal gene transfer (Hatfull and Hendrix, 2011). Together these data demonstrated that phage YA3 is a novel phage.

**TABLE 1:** Properties of YA3-related phages included in this study.

**Phage name**	**Accession**	**Genome size (bp)**	**Circular or linear**	**ORFs**	**GC content**	**tRNA**	**Coverage (%)**	**Identity (%)**	**Lysogenic or lysis**	**Classification**	**Reference**
Ab31	NC_023575.1	45,550	Circular	69	57.1	No	48	95.93	Lysogenic	*Podoviridae*	Latino et al., 2014
JBD44	NC_030929.1	49,033	Linear	80	58.5	No	14	98.17	Lysogenic	*Siphoviridae*	Magnan, 2015; Bondy-Denomy et al., 2016
PAJU2	NC_011373.1	46,872	Linear	79	56.3	No	13	88.02	Lysogenic	*Siphoviridae*	Uchiyama et al., 2009
Phi1	KT887557.1	57,218	Linear	69	57.5	No	10	84.04	ND	*Siphoviridae*	van Belkum et al., 2015
BR141b	MK511016.1	45,372	Linear	75	58.7	No	7	92.98	Lysogenic	Unclassified	Tariq et al., 2019
YMCI1/ 02/R656	NC_028657.1	60,919	Linear	113	58.7	1	6	95.70	ND	*Siphoviridae*	Jeon and Yong, 2019
D3	NC_002484.2	56,426	Linear	99	57.8	4	5	80.42	Lysogenic	*Siphoviridae*	Kropinski, 2000

*ND, not determined.*

**FIGURE 4:**
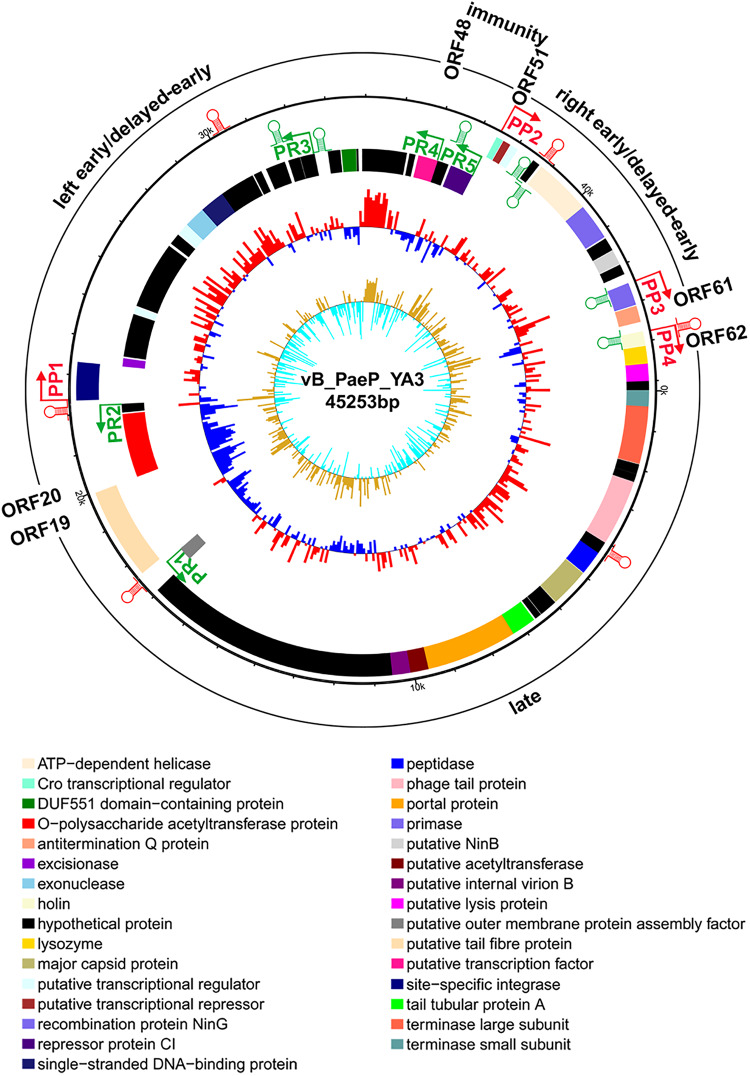
Genome organization of phage YA3. The first circles represent the predicted ORFs, where the outward ones show the forward transcription genes and the inward ones show the reversed transcription genes. A total of 65 open reading frames (ORFs) are marked with different colors according to their functions and are arranged in left early/delayed-early gene region (ORF48–ORF20), immunity region (ORF49–ORF50), right early/delayed-early gene region (ORF51–ORF61), and late region (ORF62–ORF19). The predicted positive and reverse promoters are shown as red and green arrows, respectively, on the YA3 genome. The second circle shows the G/C content. The red outward and the blue inward indicate that the G/C content of this region is high and less than the average G/C content of the whole genome, respectively. The third circle shows the GC skew.

From the multiple analyses using artemis software, blast, HHpred, and conserved domain analysis, a total of 65 putative open reading frames (ORFs) were predicted in the YA3 genome. The coding density was 1.436 genes per 1 kb, and the average gene length was 655 bp. As the YA3 genome is most related to that of phage Ab31, which is also a circular DNA, we assigned the first ORF of YA3 as a terminase small subunit like phage Ab31. The detailed information about these ORFs is shown in Figure 4 and Table 2. Of the 65 ORFs, 34 were transcribed on the positive strand, and 31 were on the reverse strand. A total of 32 gene products could be assigned to putative functions and the other 33 ORFs encode the conserved hypothetical proteins. YA3 does not contain any obvious tRNA genes or rRNA genes.

**TABLE 2:** Predicted open reading frames (ORF) in the YA3 genome.

**ORF**	**Start**	**Stop**	**Direction**	**Number of residues**	**Molecular mass (kDa)**	**Calculated isoelectric point**	**Putative function**	**Conserved protein domain superfamily**
ORF1	1	462	+	153	17.3	6.07	Terminase small subunit	HTH
ORF2	410	1876	+	488	55.5	6.61	Terminase large subunit	Terminase_6C; Terminase_6
ORF3	1888	2142	+	84	8.6	8.14	Hypothetical protein	
ORF4	2144	2350	+	68	7.5	4.64	Hypothetical protein	
ORF5	2356	4044	+	562	62.6	5.06	Phage tail protein	Head–tail_con
ORF6	4045	4356	+	103	12.2	5.21	Hypothetical protein	
ORF7	4353	4976	+	207	22.5	4.72	Peptidase	PHA00666 (putative protease)
ORF8	4989	5993	+	334	36.0	5.83	Major capsid protein	PHA00665 (major capsid protein)
ORF9	6004	6462	+	152	15.9	5.14	Hypothetical protein	PHA00664 (hypothetical protein)
ORF10	6473	6646	+	57	6.3	4.71	Hypothetical protein	
ORF11	6692	7321	+	209	23.1	4.67	Tail tubular protein A	PHA00662 (hypothetical protein)
ORF12	7318	9657	+	779	84.8	5.19	Portal protein	PHA00661 (hypothetical protein)
ORF13	9638	10117	+	159	18.5	5.24	Putative acetyltransferase	DUF2833 (protein of unknown function)
ORF14	10102	10566	+	154	16.5	9.85	Putative internal virion B	PHA00660 (hypothetical protein)
ORF15	10566	12602	+	678	73.8	5.46	Hypothetical protein	PHA00658 (putative lysin)
ORF16	12599	14521	+	640	66.6	4.66	Hypothetical protein	
ORF17	14521	17064	+	847	93.5	9.22	Hypothetical protein	PHA01972 (structural protein)
ORF18	17065	17475	−	136	14.7	5.18	Putative outer membrane protein assembly factor	
ORF19	17634	19985	+	783	82.9	5.22	Putative tail fiber protein	Phage_tailspike_middle
ORF20	19986	21914	−	642	71.4	9.05	*O*-polysaccharide acetyltransferase protein	Acyl_transf_3 (peptidoglycan/LPS *O*-acetylase OafA/YrhL)
ORF21	21960	22220	−	86	9.6	9.61	Hypothetical protein	
ORF22	22368	23342	+	324	36.5	10.10	Site-specific integrase	XerC superfamily (integrase), DNA_BRE_C superfamily
ORF23	23339	23584	−	81	9.3	10.00	Excisionase	
ORF24	23637	24089	−	150	16.7	4.96	Hypothetical protein	
ORF25	24086	24439	−	117	13.4	4.56	Hypothetical protein	
ORF26	24436	24654	−	72	8.1	9.30	Hypothetical protein	
ORF27	24651	24950	−	99	11.2	4.75	Hypothetical protein	
ORF28	24943	25161	−	72	8.1	9.85	Putative transcriptional regulator	z-alpha
ORF29	25200	25487	−	95	10.8	5.87	Hypothetical protein	
ORF30	25480	26088	−	202	21.9	4.69	Hypothetical protein	
ORF31	26081	27241	−	386	42.2	4.63	Hypothetical protein	SMC_prok_B
ORF32	27328	27501	−	57	6.4	9.69	Hypothetical protein	
ORF33	27470	27673	−	67	7.2	4.27	Hypothetical protein	
ORF34	27708	28070	−	120	13.2	10.10	Putative transcriptional regulator	
ORF35	28067	28711	−	214	24.1	4.95	Exonuclease	YqaJ (YqaJ-like viral recombinase domain)
ORF36	28708	29532	−	274	30.0	5.76	Single-stranded DNA-binding protein	ERF (DNA single-stranded annealing proteins)
ORF37	29466	30530	−	354	39.8	5.25	Hypothetical protein	PTZ00121
ORF38	30573	30821	−	82	8.8	8.59	Hypothetical protein	
ORF39	30972	31136	−	54	5.8	9.99	Hypothetical protein	
ORF40	31133	31573	−	146	16.4	4.77	Hypothetical protein	
ORF41	31680	32045	−	121	14.1	4.77	Hypothetical protein	
ORF42	32054	32494	−	146	16.1	4.88	Hypothetical protein	
ORF43	32893	33270	−	125	14.0	4.21	Hypothetical protein	
ORF44	33305	33742	−	145	16.0	4.40	DUF551 domain-containing protein	DUF551
ORF45	33751	33813	−	20	2.4	3.67	Hypothetical protein	
ORF46	33925	35274	−	449	45.6	5.37	Hypothetical protein	PRK09946 (hypothetical protein)
ORF47	35350	35532	−	60	7.3	4.94	Hypothetical protein	
ORF48	35623	36222	−	199	23.4	8.79	Putative transcription factor	
ORF49	36219	36560	−	113	12.8	9.73	Hypothetical protein	
ORF50	36638	37348	−	236	26.0	5.06	Repressor protein CI	LexA; HTH-XRE
ORF51	37449	37628	+	59	6.5	9.82	Cro transcriptional regulator	P22_Cro (DNA-binding transcriptional regulator Cro)
ORF52	37642	37842	+	66	7.7	8.11	Putative transcriptional repressor	
ORF53	37862	38065	+	67	7.6	6.25	Putative transcriptional regulator	
ORF54	38537	38731	+	64	7.2	10.28	Hypothetical protein	
ORF55	38795	40282	+	495	55.2	9.03	ATP-dependent helicase	SSL2 (superfamily II DNA or RNA helicase)
ORF56	40255	41187	+	310	34.4	8.59	Primase	COG4643 (uncharacterized domain associated with phage/plasmid primase)
ORF57	41227	41577	+	116	13.0	8.26	Hypothetical protein	
ORF58	41570	41986	+	138	15.9	6.60	Putative NinB	NinB
ORF59	41983	42246	+	87	9.8	4.96	Hypothetical protein	SH3
ORF60	42488	43069	+	193	22.1	9.87	Recombination protein NinG	NinG
ORF61	43095	43514	+	139	15.7	5.56	Putative antitermination Q protein	Phage_antitermQ
ORF62	43754	44134	+	126	13.5	7.83	Holin	Phage_holin_2_3
ORF63	44131	44583	+	150	16.2	9.35	Lysozyme	Endolysin_R21-like
ORF64	44580	45023	+	147	15.8	6.74	Putative lysis protein	Phage_lysis
ORF65	45020	45250	+	76	8.3	4.65	Hypothetical protein	

Several genes related to phage lysogeny were found in the YA3 genome, including *orf22* (site-specific integrase), *orf23* (excisionase), and *orf35* (exonuclease). Using HHpred analysis, these three lysogeny-related genes are similar with those proteins of the lambdoid phages. Site-specific integrase (ORF22) is similar with integrase of *Enterobacteria* phage lambda (100% probability) and *Enterobacteria* phage P2 (100% probability). Excisionase (ORF23) is similar with excisionase of *Enterobacteria* phage lambda (98.56% probability) and *Enterobacteria* phage P2 (98.68% probability). Exonuclease (ORF35) is similar with exonuclease of *Enterobacteria* phage lambda (100% probability). In addition, the products of the three ORFs would be proteins which possess sequence homology to CI, Cro, and Q protein of lambdoid phages after analysis using HHpred. The CI homolog (ORF50, repressor protein CI) is a 236-amino-acid protein, which is similar with the CI of *Enterobacteria* phage lambda (99.58% probability) and *Enterobacteria* phage 186 (98.67% probability), respectively. The Cro homolog (ORF51, Cro transcriptional regulator) is a 59-amino-acid protein, which is similar with the regulatory protein Cro of *Enterobacteria* phage P22 (98.76% probability), repressor protein of *Enterobacteria* phage N15 (98.95% probability), and lambda repressor Cro of *Enterobacteria* phage lambda (98.46% probability), respectively. The Q homolog (ORF61, antitermination Q protein) is a 139-amino-acid protein, which is similar with antiterminator Q protein of *Enterobacteria* phage SfI (99.8% probability). *Enterobacteria* phage SfI is known to be a member of the lambdoid family (Sun et al., 2013). N15 is a temperate virus of *E. coli* related to lambdoid phages (Ravin et al., 1999). *Enterobacteria* phage P22 is a typical member of lambdoid phages. Phage 186 and P2 form a family of phages which is similar to the lambdoids (Skalka and Hanson, 1972). CI of lambdoid phages is a transcriptional repressor that allows the virus to establish and maintain latency. CII directs the synthesis of CI repressor during the establishment phase of lysogenization through active PRE promoter (also called PE) (Kedzierska et al., 2004). Q antiterminator can recognize RNA polymerase near late promoter PR and accelerates it through a pause site (Marr et al., 2001). Furthermore, the spatial arrangement of these three genes in YA3 is identical to that of the lambda *cI*, *cro*, and *Q* gene (Liu et al., 2013). The presence of these genes strongly suggests that YA3 is lysogenic, which is similar with lambdoid phages. Meanwhile, five of the seven sequence-related phages with YA3 (Table 1) are all lysogenic. Therefore, phage YA3 is supposed to be a novel lysogenic phage of *P. aeruginosa* and a new member of the lambdoid family. It should be explored as a candidate transducing phage for *P. aeruginosa*.

To understand the transcriptional pattern of phage YA3, the promoters within its genome were searched by combining the promoter prediction softwares and manual analysis (Figure 4 and Supplementary Table S3). Four putative promoters, in front of *orf22*, *orf51*, *orf60*, and *orf62*, were identified in the positive strand. Five promoters were found in the reverse direction, located in the upstream of *orf18*, *orf21*, *orf42*, *orf48*, and *orf50*, respectively. We designated the four positive promoters as *P*P1–*P*P4 and the five reverse promoters as *P*R1–*P*R5. Two oppositely directed promoters, *P*P2 and *P*R5, were predicted between *orf50* and *orf51*, which encode CI repressor (*orf50*) and Cro transcriptional regulator (*orf51*), respectively. In lambdoid phages, CI and Cro repress each other through binding in the intergenic region and control the switch between lysogen and lysis cycle (Ptashne, 2004). The immunity region of this phage, containing the *cI* repressor gene (*orf50*), as well as putative *P*_R_ (*P*P2), *P*_L_(*P*R4), and *P*_RM_ (*P*R5) promoters, closely resembles that of lambda system and what is known about in well-studied lambda-like *P. aeruginosa* phage D3 (Kropinski, 2000). The putative *cro* gene (*orf51*) product showed a high amino acid sequence similarity (identity 66.1%, similarity 81.4%) with Cro protein of *P. aeruginosa* phage YMC11/02/R656 (Jeon and Yong, 2019). Phages lambda and 434 contain *rex* and *hex* genes, respectively, which are downstream of the *cI* gene and are transcribed from *P*_RM_ (Shkilnyj et al., 2013). ORF49 exists between the *cI* repressor gene (*orf50*) and the proposed *P*_L_ (*P*R4), which is similar with *P. aeruginosa* phage D3 (Kropinski, 2000), but *orf49* is not related to known *rex* or *hex* genes of lambdoid phages. In the classical lysogenic phages, such as lambda phage, the late cluster promoter (*P*${}_{\text{R}}^{\prime }$) exists downstream of the antitermination Q protein. Q protein binding on the *P*${}_{\text{R}}^{\prime }$ region initiates and permits the continuation of transcription through all the late genes (Deighan et al., 2008; Liu et al., 2013). Q protein encoded by *orf61* may also help RNA polymerase read through all the late genes of YA3. Consistent with this, there is also a promoter (*P*P4) downstream of the antitermination *Q* gene (*orf61*), suggesting that *orf62* is the first gene of the late region. Therefore, the YA3 genome can be divided into the immunity region (ORF49–ORF50) to repress all the gene expressions in latency, the left early/delayed-early gene region (ORF48–ORF20), the right early/delayed-early gene region (ORF51–ORF61) responsible for host interaction and DNA replication, and the late region (ORF62–ORF19) encoding the morphogenesis and lysis proteins, which is in agreement with the arrangement of classical temperate lambda-like phages (Kropinski, 2000; Marr et al., 2001).

The genome of phage YA3 was also scanned for the presence of Rho-independent terminators using ARNold^9^. The predicted Rho-independent terminators on the YA3 genome are summarized in Supplementary Table S4. The search procedure of ARNold uses two complementary programs, Erpin (Gautheret and Lambert, 2001) and RNAmotif (Macke et al., 2001). Eleven terminators can be found by both Erpin and RNAmotif programs, which were marked in Figure 4. Using RNAmotif program, we can find 10 more terminators (Supplementary Table S4). The early and late gene operons of lambdoid phages are regulated at the level of transcription termination. Lambdoid phages (*E. coli* phages lambda, 82, and 21 and *Salmonella* phage P22) encode antitermination proteins, exemplified by the N and Q proteins of phage lambda, which positively regulate the expression of the phage early and late gene operons, respectively (Guo et al., 1991; Krupp et al., 2019). RNA polymerase (RNAP) modified by these antitermination proteins transcribe through one or more termination sites. N/Nus-factor/nut RNA-modified RNAP resists pause and termination signals several kilobase pairs downstream of the modification site (Mason et al., 1992). Several terminators (tRf9, tRr3, and tRr4) locate several kilobase pairs downstream of *P*_R_ (*P*P2) or *P*_L_(*P*R4), which may be read through by RNAP in the presence of anti-termination factors such as N. The N protein homology was not found in the YA3 genome. A putative transcription factor (ORF48) downstream of *P*_L_ (*P*R4), which takes the place of N, may function as an antiterminator to modify RNAP and transcribe through the terminators downstream of *P*_R_ (*P*P2), *P*_L_(*P*R4). Q protein homology (antitermination Q protein encoded by *orf61*) was found in YA3, which is sharing <50% identity with its equivalents from all the other phages using blastp. After analysis by HHpred, we found that this antitermination Q protein is similar with the antiterminator Q protein of lambdoid phage SfI (99.8% probability). The antitermination Q protein of lambda phage binds to the RNAP near the phage late gene promoter *P*_R_′, which allows it to pass through tR′ on late operon (Grayhack et al., 1985; Shi et al., 2019). The rho-independent terminator (tRf10) at 224 nucleotides downstream of the late operon promoter (*P*P4) is similar with the terminator tR′ downstream of *P*${}_{\text{R}}^{\prime }$ in lambdoid phages. The antitermination Q protein of YA3 may bind to RNAP near the phage late gene promoter (*P*P4) and help RNAP read through terminator (tRf10) on late operon. We speculate that phage YA3 has a genetic organization and an antitermination control mechanism similar to those of lambdoid phages.

### Comparison of the Genomes of YA3 With Ab31

The genome of phage Ab31, which is most genetically closely related to YA3, was reported to have structural genes related to lytic phage and regulatory genes similar to lysogenic phage (Latino et al., 2014). To further investigate the YA3 genome, we then compared the genome organizations and protein sequence similarities between YA3 and Ab31 using BLAST and EMBOSS Needle tool.

**FIGURE 5:**
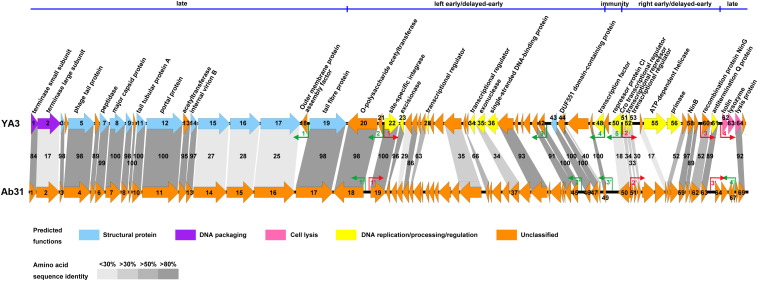
Genome comparison of phage YA3 and vB_PaeP_Tr60_Ab31 (Ab31). The predicted proteins encoded by the YA3 genome, except the hypothetical proteins, are shown at the top. Arrows represent predicted CDSs, with the direction of the arrow indicating the transcription direction. The colors indicate different functional groups of gene products: light blue, structural protein; purple, DNA packaging; pink, cell lysis; yellow, DNA replication/processing/regulation; and orange, unclassified. The immunity region (ORF49–ORF50), left early/delayed-early gene region (ORF48–ORF20), right early/delayed-early gene region (ORF51–ORF61), and late region (ORF62–ORF19) of YA3 were marked and separated by a blue line at the top of this figure. The homologous ORFs are connected by the different gray-shaded regions, and the percentages of amino acid sequence identities are shown in the shaded regions. The predicted positive and reverse promoters are shown as red and green arrows, respectively, on their genomes.

In agreement with the fact that these two phages have identical morphology, the structure genes on late cluster are very similar (Figure 5). A total of 18 of the 23 YA3 proteins have homologs in Ab31 phage genome on late cluster, among which 14 are above 80% identical to the corresponding proteins of Ab31, such as major capsid protein, phage tail protein, and tail tubular protein A. Three hypothetical proteins, gp15, gp16, and gp17, on late cluster show considerably low identity (20–30%) with the homologs that are putative structural lysozyme, hypothetical protein, and hypothetical protein in Ab31, respectively. The terminase large subunit of YA3 (gp2) and Ab31 (gp2) also share very low homology (identity 16.6%, similarity 25.9%) on late cluster. The terminase large subunit of phage is reported to participate in DNA cleavage after packing into the head during phage replication (Rao and Feiss, 2008). Therefore, phage YA3 and Ab31 may have different packaging pathways. Three proteins in the beginning of late cluster, gp62 (holin), gp63 (lysozyme), and gp65 (hypothetical protein), have no homolog in Ab31. Of these genes, *orf62* and *orf63* belong to the predicted lysis module of YA3 genome, which contains *orf62*, *orf63*, and *orf64*. The results of the HHpred and conserved domain analysis suggested that gp62 has one transmembrane domain and is believed to encode the holin. This protein sequence has not been found in the genomes of other phages. *Orf63* encodes the lysozyme, which belongs to the Lyz_like superfamily and hits the endolysin_R21_like domain. This protein only shows significant similarity with the lytic enzyme of *Pseudomonas* phage phi3 (YP_009276446.1, identity 61.8%, similarity 74.7%) but does not find homolog in Ab31 genome. *Orf64* is annotated to be a putative lysis protein belonging to the Phage_lysis superfamily, which is highly homologous with Ab31’s lysis protein (YP_009007136.1, identity 91.8%, similarity 94.6%). Hence, the lysis module of phage YA3 is different from Ab31’s, indicating a novel lysis mechanism of this phage.

The early/delayed-early genes’ functions of phage YA3 are poorly known. Less than half of the genes (17/40) are assigned with predicted functions, and the other genes are all hypothetical. Besides that, the early/delayed-early regions of phage YA3 and Ab31 are highly variable (Figure 5). Just 60% of the early/delayed-early protein homologs (25/40) of YA3 are found in Ab31 genome. The numbers of homologs that exhibit identity of above 50, 30–50, and below 30% are 17, 6, and 2, respectively. The conserved proteins (identity above 50%) include *O*-polysaccharide acetyltransferase protein (gp20), site-specific integrase (gp22), excisionase (gp23), NinB (gp58), NinG (gp60), antitermination Q protein (gp61), and some hypothetical proteins (gp25, gp27, gp32, gp37, gp41-44, gp47, gp57, and gp59). Eight less-conserved genes (identity below 50%) are predicted to encode four hypothetical proteins (gp24, gp31, gp45, and gp54), exonuclease (gp35), Cro transcriptional regulator (gp51), putative transcriptional repressor (gp52), and putative transcriptional regulator (gp53), respectively. In addition, there are 15 early/delayed-early genes unique in the YA3 genome. Except for nine hypothetical proteins, the single-stranded DNA-binding protein (gp36), ATP-dependent helicase (gp55), primase (gp56), putative transcriptional regulators (gp28 and gp34), and putative transcription factor (gp48) are present only in phage YA3. The homology of the CI (gp50) and Cro (gp51) analogs of these two phages are also relatively low (identity 18.0%, similarity 31.5% and identity 34.3%, similarity 47.1%, respectively), which is consistent with their poor sequence conservation between different phages (Dubrava et al., 2010; Hall et al., 2019). As for the promoters, we found three positive promoters in front of *orf19*, *orf51*, and *orf64* (named *P*P1′–*P*P3′) and four reverse promoters upstream of *orf18*, *orf4*5, *orf49*, and *orf67* (named *P*R1′–*P*R4′) in the Ab31 genome (Figure 5). Only four of these promoters, *P*P1′, *P*P2′, *P*R1′, and *P*R2′, are corresponding with the YA3 promoters. These results indicated the significant difference between the regulation patterns of YA3 and Ab31.

In conclusion, although YA3 has the closest relationship with Ab31, the identity of their genomes is not high. Furthermore, there are significant differences between them in many important processes, including the lysis pathways and the transcriptional regulation patterns. It is well known that there are many unknown open reading frames termed ‘ORFans’ in phage genomes, especially in the early/delayed-early regions, and they are also less conserved between phages (Yin and Fischer, 2008). Several ORFans have been proven to mediate crucial processes such as host takeover and transcriptional regulation during phage infection (Roucourt and Lavigne, 2009; De Smet et al., 2017; Boon et al., 2019). Therefore, revealing the functions of ORFans in phage YA3 genome is believed to discover some distinct protein functions.

### Structural Proteins of YA3

**FIGURE 6:**
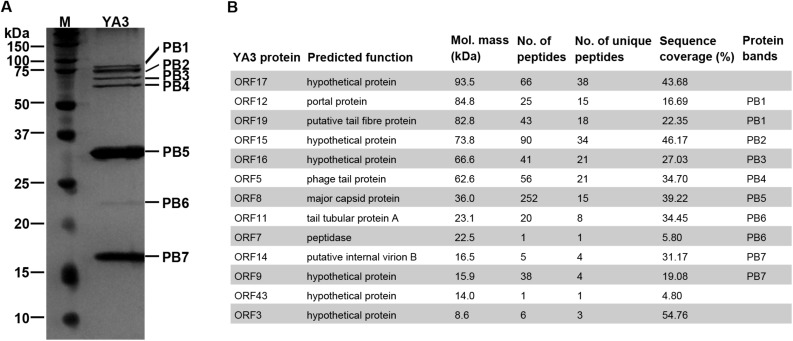
Identification of YA3 virion structural proteins. **(A)** The structural proteins of purified YA3 particles were separated by SDS-PAGE and stained with silver. The molecular mass markers are shown on the left. **(B)** The structural proteins of YA3 as detected by mass spectrometry. The molecular mass was calculated from the gene sequence. The number of identified peptides and unique peptides in each protein and the corresponding protein sequence coverage are also indicated.

To investigate the structural proteins of YA3 virions, SDS-PAGE was firstly performed to separate the purified YA3 sample. As shown in Figure 6A, seven obvious bands were observed, corresponding to molecular masses of 16–85 kDa. To further uncover the related genes with its structure, mass spectrometry was carried out using the phage YA3 particles digested with trypsin. Using all the YA3-encoded proteins as the database, 13 structural proteins of YA3 were detected (Figure 6B). The detailed results from mass spectrometry including peptide sequences are provided in Supplementary Data S1. When comparing with the SDS-PAGE result, 10 detected proteins could match the protein bands (Figure 6). Of note is that three hypothetical proteins (gp17, gp43, and gp3) were not visible because of their low content in the YA3 virions. The dominant protein band PB5 in SDS-PAGE is corresponding to *orf8*, which is considered to encode the major capsid protein.

Among these proteins identified by mass spectrometry, 12 are all located in the late region, except for a hypothetical protein encoded by *orf43*. The known structural proteins of phages including gp8 (major capsid protein), gp19 (tail fiber protein), gp5 (phage tail protein), gp11 (tail tubular protein A), gp14 (internal virion B), and gp12 (portal protein) were all confirmed. Gp7, the peptidase, was also found in the YA3 particles. Peptidases, or called proteases, have been found in phage virions to promote the head maturation of some other phages such as phage lambda, T4, and *Salmonella* phage SPN3US (Medina et al., 2010; Fokine and Rossmann, 2016; Ali et al., 2017; Weintraub et al., 2019). However, the functions of the other six genes encoding hypothetical proteins are unknown. Studying how these structural proteins function, especially the six hypothetical proteins, may increase our knowledge of the morphological composition of phages.

Besides that, the 13 structural-related genes all exist in the phage Ab31 genome (Figure 5). Ten of these 13 genes are highly conserved, with identity above 97%, in agreement with the fact that phage YA3 and Ab31 have the same morphology. The other three genes (*orf15–orf17*), which are all hypothetical proteins and adjacently arranged, are significantly variable, showing the identity to be less than 30%. Meanwhile, the homologs of these three genes are not found in any other phage throughout blastx in NCBI. These results indicated that the morphological compositions of phage YA3 and Ab31 are similar but still have slight differences which might be caused by the three hypothetical proteins (gp15–gp17). Exploring these three proteins’ functions may reveal the morphology difference between the two phages.

### Integration Site of YA3 in PA14 Genome

**FIGURE 7:**
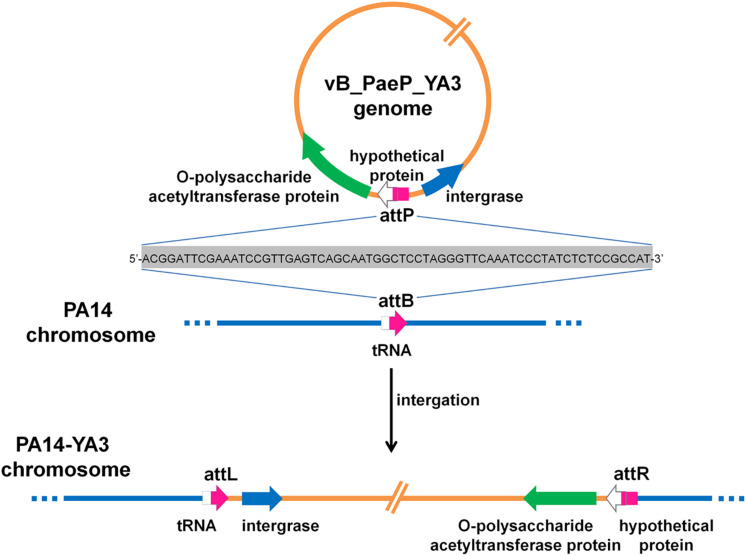
The schematic form of phage YA3 integration into the chromosome of *P. aeruginosa* PA14. The peptidoglycan *O*-acetyltransferase gene, hypothetical protein, and integrase gene of YA3 as well as the tRNA gene of PA14 are indicated by large arrows. The attP is used as the attachment site to bind with attB on the PA14 genome for integration. attL and attR represent the left and the right attachment sites of the lysogenic PA14-YA3 strain, respectively. The attP, attB, attL, and attR sites are marked in pink, and their 64-bp direct repeat sequences are also shown.

To identify the integration site of YA3, the YA3-lysogenized PA14 strain, named PA14-YA3, was isolated (see details in section “Materials and Methods”). After at least three consecutive subcultures and PCR identifications, the lysogenization of YA3 to PA14 was still stable (data not shown). Then, we extracted the PA14-YA3 genomic DNA and performed draft genome sequencing. The YA3 genome sequence was then searched in the PA14-YA3 draft genome by two-sequence alignment. The result showed that the YA3 genome sequence was integrated into the PA14 genome using the region 4553037–4552974 of PA14 genome as the attB site and 22196–22259 of phage YA3 genome as the attP site, and the sequences of attB and attP sites are just the same (Figure 7). The attB site consists of the last 63 bp and the following 1 bp of the serine tRNA gene (PA14_RS20820) of PA14, and the attP site contains the beginning 25 bp and the 39 bp upstream of *orf21* (hypothetical protein). As shown in Figure 7, in the lysogenic strain PA14-YA3, the reconstituted serine tRNA gene (PA14_RS20820) of PA14 and *orf21* of YA3 are still intact. The integration sites and the sequences of phage YA3 and Ab31 are identical (Latino et al., 2014), indicating the conserved integration mechanism between the two phages. In addition, the fact that the target site of integration overlaps with a putative tRNA gene of the host genome is common, which may help increase the number of different host chromosomes where they can integrate (Williams, 2002).

### Phylogenetic Analysis of YA3 and Related Phages

**FIGURE 8:**
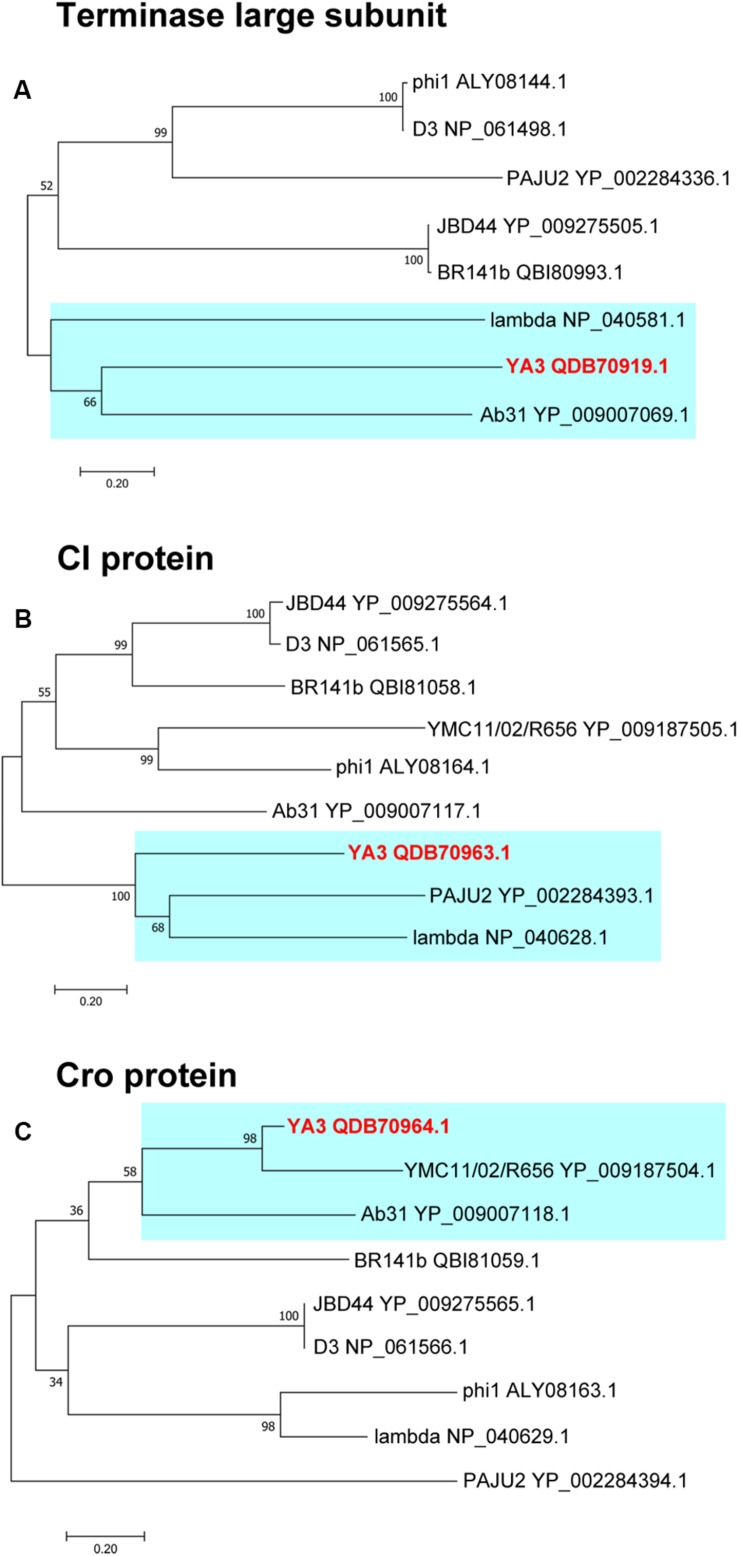
Phylogenetic analysis of YA3 and related phages. The terminase large subunits **(A)**, CI proteins **(B)**, and Cro proteins **(C)** of selected phages were compared using the ClustalW program, and the phylogenetic tree was generated using the neighbor-joining method with 1,000 bootstrap replicates. The accession numbers of phages encoding particular terminase large subunits, CI proteins, and Cro proteins are given following the names of the phages. The branches of phage YA3 are marked in cyan.

To further explore the evolutionary position of YA3, the phylogenetic analysis of YA3, the seven related phages (Table 1), and the classical lambda phage were analyzed using the neighbor-joining method. Phage terminase large subunit (TerL) is a relatively conserved protein and is mainly used as a phylogenetic marker in the comparative analysis of phage genomes (Feiss and Rao, 2012). Hence, we firstly built the phylogenetic tree based on TerL of these phages. As expected, the result suggested that phage YA3 and Ab31 were the most closely related (Figure 8A). Besides that, the terminase large subunits of phage lambda and YA3 were classified in the same branch, indicating that they are quite related. This result was surprising because the genome coverage of the lambda phage and YA3 is extremely low (<1%), less than the other seven related phages. We then compare the two lysogenic regulatory proteins CI and Cro of these phages to explore more information about their phylogenetic relationships. As shown in Figures 8B,C, the results were more complicated. The CI homology of YA3 (gp50) is more related with those of PAJU2 and lambda phage, while its Cro protein (gp51) is more similar with those of YMCI1/02/R656 and Ab31 phage. The three considerably different phylogenetic trees suggested that YA3 and the seven *Pseudomonas* phages are not very relevant, and their evolutional relationships are far more complicated than we expected. Combining the three evolutionary analyses, as well as some identical properties including the *Podoviridae* morphology and circular genome, we concluded that phage YA3 is most related to Ab31. Moreover, despite the low homology of genomes of phage YA3 and lambda, some conserved proteins such as terminase large subunit and CI protein (gp50) are similar between them, indicating that YA3 belongs to the member of lambda-like phages.

Although all the proteins encoded by YA3 show no similarity to the proteins of T7 in a BLASTP search, HHpred analysis reveals a similarity on some proteins. The terminase large subunit (ORF2, 100% probability), phage tail protein (ORF5, 100% probability), tail tubular protein A (ORF11, 100% probability), portal protein (ORF12, 100% probability), and primase (ORF56, 99.88% probability) of YA3 are similar with their corresponding proteins of T7. YA3 is partially related to T7. Because YA3 and T7 are both *Podoviridae*, they may have some similar structural proteins. Still there are also many differences between their structural proteins. The phage T7 is a lytic phage; however, the phage YA3 is temperate. For all these reasons, the difference between YA3 and T7 phage is relatively large. Phage YA3 is an unusual phage, which possesses features of both temperate lambdoid phages and lytic T7 phage. In this, it resembles the most genetically closely related phage Ab31 (Latino et al., 2014).

In addition, infection of *P. aeruginosa* strain PAO1 (serotype O5) by *Pseudomonas* phage D3 (one of the homologous phages of YA3) causes the serotyping switch of *P. aeruginosa* serotype O5 to O16. Three adjacent gene expression products of phage D3, an *O*-acetylase, a b-polymerase, and an inhibitor of the host a-polymerase, could be involved in the serotype conversion (Kropinski, 2000). We found that the *O*-polysaccharide acetyltransferase protein (ORF20) of phage YA3 and the *O*-acetylase of phage D3 have some homology (identity 27.5%, similarity 44.5%) after analysis using the EMBOSS Needle tool at EMBL-EBI. Several serotype conversion bacteriophages of *Shigella flexneri*, SfI, SfII, SfIV SfV, SfX, and Sf6, have previously been isolated and studied. The genes involved in serotype conversion in these *Shigella* phages are located adjacent to the int attP region. The glucosyltransferase and/or acetyltransferase genes encoded by these *Shigella* phages are responsible for the modification of the O-antigen of their host strains (Jakhetia et al., 2013). The *O*-polysaccharide acetyltransferase protein (ORF20) of phage YA3 is not similar to the genes involved in serotype conversion in *Shigella* phages, but the *O*-polysaccharide acetyltransferase protein (ORF20) of phage YA3 is very close to the attP site of YA3, which is similar with the genes involved in *Shigella* serotype conversion. Therefore, we concluded that phage YA3 may be potentially a serotype-converting phage.

All the results demonstrated that the evolutionary relationships between lysogenic phages and even their hosts are far more complicated than those of lytic phages, and more in-depth studies are needed to better understand this question.

## Conclusion

Here phage YA3 was characterized and completely sequenced as a novel lambda-like temperate phage of *P. aeruginosa*. As has been observed for other temperate bacteriophages, they may be virulence-enhancing elements or involved in horizontal gene transfer (HGT) (Wagner and Waldor, 2002). The detailed genome analysis of the phage provides us with crucial molecular insight into the differences between the temperate phages of *P. aeruginosa* and may also contribute to research into not only the evolution of *P. aeruginosa* temperate phages but also the genomic diversity of *P. aeruginosa* due to phage-derived HGT. In addition, a further study of the genes’ function of phage YA3, especially the functions of unknown genes, may shed light on phage evolution and phage–host interactions and may also help to develop new phage therapy strategies for *P. aeruginosa*.

## Data Availability Statement

The nucleotide sequence of phage YA3 genome has been deposited in GenBank under accession number MK819239.

## Ethics Statement

The studies involving human participants were reviewed and approved by Ethics Committee of the Second Affiliated Hospital of Nanjing Medical University. The patients/participants provided their written informed consent to participate in this study. Written informed consent was obtained from the individual(s) for the publication of any potentially identifiable images or data included in this article.

## Author Contributions

XY and XL designed the study. XY, JX, YG, and RZ performed the experiments. XY and XL analyzed the data and wrote the manuscript. YZ provided the clinical isolates of *P. aeruginosa*. All authors contributed to the manuscript revision and read and approved the submitted version.

## Conflict of Interest

The authors declare that the research was conducted in the absence of any commercial or financial relationships that could be construed as a potential conflict of interest.

## Footnotes

1 http://www.sanger.ac.uk/science/tools/artemis

2 http://blast.ncbi.nlm.nih.gov/Blast.cgi

3 http://www.ncbi.nlm.nih.gov/Structure/cdd/wrpsb.cgi

4 http://lowelab.ucsc.edu/tRNAscan-SE

5 http://www.cbs.dtu.dk/services/RNAmmer/

6 https://galaxy.bio.di.uminho.pt/?tool_id=get_proms&version=0.1.0&__identifer=4u05obc3o5w

7 http://www.fruitfly.org/seq_tools/promoter.html

8 http://rssf.i2bc.paris-saclay.fr/toolbox/arnold/

9 http://rssf.i2bc.paris-saclay.fr/toolbox/arnold/
