# Supplementary material for: Molecular Characterization and Comparative Genomic Analysis of vB_PaeP_YA3, a Novel Temperate Bacteriophage of Pseudomonas aeruginosa
Source: Front Microbiol. 2020 Jun 3;11:947. doi: 10.3389/fmicb.2020.00947 (PMC7326022; doi:10.3389/fmicb.2020.00947)
Supplement: Supplementary file 2 [file Table_1.docx]

**Table S1. The host range of phage YA3.**

| Strains | Resistance | Source | Lysed or not^a^ |
| --- | --- | --- | --- |
| *P. aeruginosa* PA14 |  | Standard Laboratory Reference Strain | + |
| *P. aeruginosa* PAO1 |  | Standard Laboratory Reference Strain | - |
| *Escherichia coli*  K-12 MG1655 |  | Standard Laboratory Reference Strain | - |
| *P. aeruginosa 2320* | Amikacin, minocycline, ticarcillin/clavulanic acid, aztreonam, piperacillin, ceftazidime, ticarcillin | Sputum, Male,86^#^ | - |
| *P. aeruginosa 2321* | Minocycline, ticarcillin/clavulanic acid, aztreonam, piperacillin, ceftazidime, ticarcillin | Wound secretion, Male, 48^#^ | - |
| *P. aeruginosa 2324* | Minocycline, ticarcillin/clavulanic acid, ticarcillin | Wound secretion, Male, 71^#^ | - |
| *P. aeruginosa 2325* | Minocycline | Sputum, Female,65^#^ | - |
| *P. aeruginosa 2357* | Minocycline, ticarcillin/clavulanic acid, ticarcillin | Sputum, Male, 58^#^ | - |
| *P. aeruginosa 2372* | Ampicillin, cefotetan, ceftriaxone, cotrimoxazole, ampicillin/sulbactam, cefazolin | Blood, Female, 77^#^ | - |
| *P. aeruginosa 2382* | Amikacin, cefepime, levofloxacin, piperacillin, ceftazidime, ticarcillin, gentamicin, aztreonam, imipenem, meropenem, cefoperazone/sulbactam, ticarcillin/clavulanic acid, piperacillin/tazobactam | Sputum, Male, 87^#^ | - |
| *P. aeruginosa 2383* | Ticarcillin/clavulanic acid, imipenem, aztreonam, ceftazidime, ticarcillin, levofloxacin, cefoperazone/sulbactam | Sputum, Male, 82^#^ | - |
| *P. aeruginosa 2384* | Ticarcillin, imipenem, ticarcillin/clavulanic acid | Sputum, Female, 69^#^ | - |
| *P. aeruginosa 2387* | meropenem, ticarcillin/clavulanic acid, aztreonam, levofloxacin, ticarcillin, imipenem | Sputum, Male, 69^#^ | - |
| *P. aeruginosa 2388* | Amikacin, cefepime, levofloxacin, gentamicin, imipenem, cefoperazone/sulbactam, piperacillin/tazobactam, ampicillin, cefoxitin, ciprofloxacin, tigecycline, cotrimoxazole, amoxicillin/clavulanic acid, cefazolin, ceftriaxone, tobramycin, nitrofurantoin | Urine, Male, 81^#^ | - |
| *P. aeruginosa 2395* | Ampicillin, cefotetan, ceftriaxone, cotrimoxazole, ampicillin/sulbactam, cefazolin | Blood, Female, 67^#^ | - |
| *P. aeruginosa 2399* | Imipenem | Blood, Female, 73^#^ | - |
| *P. aeruginosa 2401* | Amikacin, ticarcillin, gentamicin, imipenem, ticarcillin/clavulanic acid | Sputum, Male, 86^#^ | - |
| *P. aeruginosa 2541* | Amikacin, cefepime, levofloxacin, piperacillin, ceftazidime, ticarcillin, gentamicin, aztreonam, imipenem, meropenem, cefoperazone/sulbactam, ticarcillin/clavulanic acid, piperacillin/tazobactam | Sputum, Male, 50^#^ | - |
| *P. aeruginosa 2558* | Ticarcillin, ticarcillin/clavulanic acid | Sputum, Female, 64^#^ | - |
| *P. aeruginosa 2579* | Ticarcillin, imipenem, ticarcillin/clavulanic acid | Urine, Female, 67^#^ | - |
| *P. aeruginosa 2612* | Imipenem | Sputum, Female, 61^#^ | - |
| *P. aeruginosa 2619* | Levofloxacin, piperacillin, ticarcillin, gentamicin, imipenem, meropenem, ticarcillin/clavulanic acid | Sputum, Male, 81^#^ | - |
| *P. aeruginosa 1609443* | Levofloxacin, ticarcillin, gentamicin, imipenem, ticarcillin/clavulanic acid | n.d.^b^ | - |

a. +, phage-susceptible; -, phage-resistant;

b. n.d., no data available;

^#^ . numbers in the column “source” represent “the age of the individual”.
