# Supplementary material for: Molecular Characterization and Comparative Genomic Analysis of vB_PaeP_YA3, a Novel Temperate Bacteriophage of Pseudomonas aeruginosa
Source: Front Microbiol. 2020 Jun 3;11:947. doi: 10.3389/fmicb.2020.00947 (PMC7326022; doi:10.3389/fmicb.2020.00947)
Supplement: Supplementary file 3 [file Table_2.docx]

**Table S2. Primers and their respective product sizes.**

| Amplicon region | Primer name | Primer sequence | Expected size of the PCR amplicon (bp) |
| --- | --- | --- | --- |
| 531-1911 | YA3-531F | CGCTGTCGTGAGGGTGGTA | 1381 |
|  | YA3-1911R | TGTTCGTGAGGGAGTCTGG |  |
| 5630-7048 | YA3-5630F | GGGTCACTGGGATGGACT | 1419 |
|  | YA3-7048R | GGTCAGCTTCGTGGTGC |  |
| 20622-21991 | YA3-20622F | ATCGCCAGGCACGTCAG | 1370 |
|  | YA3-21991R | TTTGGAGTTCAGCAGCTTTC |  |
| 27019-28404 | YA3-27019F | CGCCGATGATACGACTGG | 1386 |
|  | YA3-28404R | GGCCCTTGAAAGACCTGAA |  |
