# Supplementary material for: Molecular Characterization and Comparative Genomic Analysis of vB_PaeP_YA3, a Novel Temperate Bacteriophage of Pseudomonas aeruginosa
Source: Front Microbiol. 2020 Jun 3;11:947. doi: 10.3389/fmicb.2020.00947 (PMC7326022; doi:10.3389/fmicb.2020.00947)
Supplement: Supplementary file 4 [file Table_3.docx]

**Table S3. Predicted promoters in YA3 genome.**

| Name | Strand | Positions | Sequence (5’-3’) | ORF regulated |
| --- | --- | --- | --- | --- |
| *P*P1 | + | 22208-22257 | TCCGTTGAGTCAGCAATGGCTCCTAGGGTTCAAATCCCTATCTCTCCGCC | ORF22 |
| *P*P2 | + | 37383-37432 | TTTCTTTGCCTTGCACAAAATAGTGTAAGTATGCTTTCATAACGACGTGT | ORF51 |
| *P*P3 | + | 42341-42390 | CACTTGGCAGGGCAACTGCGGTGGCTGGGTGGAATCGGTTGAGGTCGATC | ORF60 |
| *P*P4 | + | 43531-43580 | TAATAGTTGACCGTGTTAACTCGAACATATAGGATTTATGGAAGATTGCG | ORF62 |
| *P*R1 | - | 17501-17550 | AACCGTTCTTATTGCTAGATTGATGACTTGGGTATACCTTCGAGCGGTCC | ORF18 |
| *P*R2 | - | 22353-22385 | TTTCTCGAAGTAAGCCATGCGGCGAACTTTAGT | ORF21 |
| *P*R3 | - | 32854-32903 | GCATCAACTGACTTCCCCGGCAAGGACGCCACCCTTTAATGGGGATGACT | ORF42 |
| *P*R4 | - | 36530-36579 | TGTAAGAGGACTTGCATCCATGTGTAAGCATGCTTATAGTTCACTCAACG | ORF48 |
| *P*R5 | - | 37400-37449 | TGTGCTTTCTCCTGAAGACACGTCGTTATGAAAGCATACTTACACTATTT | ORF50 |
