# Supplementary material for: Molecular Characterization and Comparative Genomic Analysis of vB_PaeP_YA3, a Novel Temperate Bacteriophage of Pseudomonas aeruginosa
Source: Front Microbiol. 2020 Jun 3;11:947. doi: 10.3389/fmicb.2020.00947 (PMC7326022; doi:10.3389/fmicb.2020.00947)
Supplement: Supplementary file 5 [file Table_4.docx]

**Table S4. Predicted Rho-independent terminators in YA3 genome.**

| Erpin or RNAmotif | Name | Strand | Positions | Sequence (5’-3’) |
| --- | --- | --- | --- | --- |
| Both | tRf1 | + | 4122-4166 | GAAGTCTCAGATGGCGACCCTAAACGGTCGCCGTTTTGTTTGGGA |
| RNAmotif | tRf2 | + | 6643-6681 | GTAACACCTCAGGGCCCTTCGGGGCCCTTTTCTATTTCC |
| RNAmotif | tRr1 | - | 10808-10844 | GGTTACATTCAGGGCGGCGCCGCCCTTTTTTGTCATA |
| RNAmotif | tRr2 | - | 11324-11359 | CGGCGAAGAGCGGCCATCTCGGCCTTTTGTTGAAGC |
| RNAmotif | tRf3 | + | 13953-13989 | AAATTCCGGCGCGGTTCATGGACCGTATTGCTTCACG |
| Both | tRf4 | + | 17580-17619 | CCCCCAGAGAACCCCGCCTAGTGCGGGGTTTTCGCATTTC |
| Both | tRf5 | + | 22255-22308 | GCCATATACAAGGGCTCCAGAGGTTCACGCTTCTGGAGTCCTTTTCTTTTGGGG |
| RNAmotif | tRf6 | + | 29623-29669 | TCGCGTTCGATGCGGGCCTGCTCATCGGCCTGCcgTTTGCGTTCTGC |
| Both | tRf7 | + | 30482-30528 | GAGCGGTTTCTTTGGCTGGTACTTCGACCAGTTGATTTTCTGCGGAC |
| Both | tRr3 | - | 31617-31667 | ACCTGAAAGAGGCCGCCAAAGTCCTGATCTTCGGCGGCTTTTTTGTGGGCG |
| RNAmotif | tRf8 | + | 31869-31906 | CGTAAACTCAGGGCCGATAAAGGGCCTTTCTTTCCGAT |
| Both | tRr4 | - | 32549-32589 | ACCCCTCATTAGCCCGGCAAGTCCGGGCaTTTTTTCGCCTG |
| Both | tRr5 | - | 36591-36630 | GTCAGAAAGTAGCCCGCTATAAGCGGGCTTTCTTTTGCCC |
| Both | tRr6 | - | 38503-38539 | CATCTAACTCACCCCTGGTCGGGGcTTTTCCTGGCTT |
| Both | tRr7 | - | 38727-38782 | TCTCTGAGCAACGCCCCGTTCTGTCCTCGCCGACTGCGGGGCGTTTTTGTTTCAGC |
| Both | tRf9 | + | 38729-38784 | TGAAACAAAAACGCCCCGCAGTCGGCGAGGACAGAACGGGGCGTTGCTCAGAGAGC |
| RNAmotif | tRr8 | - | 40360-40406 | AAGCGGAAGCGGTCCTTTCCACCACAGAGAGGGCaTTCTGTGTGCTT |
| Both | tRr9 | - | 42604-42644 | CGATGGCCTTCCGCGCCGGCTTGGCGTGcTTGTCCTTGATG |
| Both | tRr10 | - | 43801-43846 | AGAAACGAAAAAGCCCAGCGCTAGGCTGGGCTcTGAATTATTGGAG |
| Both | tRf10 | + | 43805-43846 | AATAATTCAGAGCCCAGCCTAGCGCTGGGCTTTTTCGTTTCT |
| RNAmotif | tRf11 | + | 43851-43893 | CTCCCAGCGCATGCCCGCAGCCGCGCGGGCGTTTTATTCCTTC |
